# The effectiveness of peer health coaching in improving glycemic control among low-income patients with diabetes: protocol for a randomized controlled trial

**DOI:** 10.1186/1471-2458-11-208

**Published:** 2011-04-01

**Authors:** Amireh Ghorob, Maria Mercedes Vivas, Diana De Vore, Victoria Ngo, Thomas Bodenheimer, Ellen Chen, David H Thom

**Affiliations:** 1 Department of Family and Community Medicine, University of California, San Francisco (UCSF), 995 Potrero Ave, Building 80/83, San Francisco, CA 94110, USA

## Abstract

**Background:**

Although self-management support improves diabetes outcomes, it is not consistently provided in health care settings strained for time and resources. One proposed solution to personnel and funding shortages is to utilize peer coaches, patients trained to provide diabetes education and support to other patients. Coaches share similar experiences about living with diabetes and are able to reach patients within and beyond the health care setting. Given the limited body of evidence that demonstrates peer coaching significantly improves chronic disease care, this present study examines the impact of peer coaching delivered in a primary care setting on diabetes outcomes.

**Methods/Design:**

The aim of this multicenter, randomized control trial is to evaluate the effectiveness of utilizing peer coaches to improve clinical outcomes and self-management skills in low-income patients with poorly controlled diabetes. A total of 400 patients from six primary health centers based in San Francisco that serve primarily low-income populations will be randomized to receive peer coaching (n = 200) or usual care (n = 200) over 6 months. Patients in the peer coach group receive coaching from patients with diabetes who are trained and mentored as peer coaches. The primary outcome is change in HbA1c. Secondary outcomes include change in: systolic blood pressure, body mass index (BMI), LDL cholesterol, diabetes self-care activities, medication adherence, diabetes-related quality of life, diabetes self-efficacy, and depression. Clinical values (HbA1c, LDL cholesterol and blood pressure) and self-reported diabetes self-efficacy and self-care activities are measured at baseline and after 6 months for patients and coaches. Peer coaches are also assessed at 12 months.

**Discussion:**

Patients with diabetes, who are trained as peer health coaches, are uniquely poised to provide diabetes self management support and education to patients. This study is designed to investigate the impact of peer health coaching in patients with poorly controlled diabetes. Additionally, we will assess disease outcomes in patients with well controlled diabetes who are trained and work as peer health coaches.

**Trial Registration:**

ClinicalTrials.gov identifier: NCT01040806

## Background

The incidence and prevalence of diabetes continues to increase globally. It is estimated that 285 million people live with diabetes worldwide, and by 2030, that number will surge to 439 million [1]. In the United States, diabetes affects approximately 24 million people and 57 million more have pre-diabetes [2]. Minorities and low-income populations carry a disproportionate burden of the disease. The prevalence of diagnosed diabetes is twice as high in non-Hispanic blacks and in Mexican Americans as it is for non-Hispanic whites [3]. Barriers to accessing the health care system compound these disparities. Latinos, African American and Asians are less likely to have had a health care visit during the previous year than are whites [4]. There is therefore an urgent need to find innovative and effective solutions to help people, especially low-income populations, successfully manage their diabetes.

One solution is to train personnel to deliver self-management support to patients with diabetes. Those trained to work with patients collaboratively to provide this support are known as health coaches. The Institute of Medicine defines self-management support as "the systematic provision of education and supportive interventions to increase patients' skills and confidence in managing their health problems, including regular assessment of progress and problems, goal setting, and problem-solving support" [5]. Self-management support involves 7 essential activities:

1) Giving information

2) Teaching disease-specific skills

3) Negotiating healthy behavior change

4) Providing training in problem-solving skills

5) Assisting with the emotional impact of having a chronic condition

6) Providing regular and sustained follow-up

7) Encouraging active participation in the management of the disease

Self-management support, which can also be called health coaching, has been shown to improve glycemic control in patients with diabetes [6]. However, most medical practices have failed to provide self-management support due to lack of personnel with protected time to provide these services. To address this problem, *patients *can be trained as peer coaches to provide self-management support to other patients.

Peer coaches, because they experience the challenges of living with diabetes, are prime candidates to offer practical and emotional support to engage and motivate other patients in the day-to-day management of their chronic condition. One example of peer coaching is the work of Latino Health Access, a non-profit organization located in Santa Ana, California, which has been training and working with peer coaches (called promotores) for 15 years. The promotores have assisted over 15,000 mostly uninsured Latino patients with diabetes to understand their disease, adhere to their medications, and navigate the health systems in which they receive their care. Another example is Project Dulce, initiated in 1997 in San Diego. Project Dulce trains patients with diabetes to provide coaching, and has assisted thousands of mostly Latino low-income patients at community clinic and university health system sites. Project Dulce has published results showing significant improvements in diabetes outcomes compared with usual care, but the intervention included RN care management in addition to peer coaching, so it is not known whether the peer coaching alone improved outcomes [7].

Although peer coaching is gaining attention as a low-cost strategy for chronic disease management, few well-designed studies have significantly shown that peer coaching improves chronic disease care. Heisler et al. showed both statistically and clinically significant reductions in HbA1c for male veterans assigned a peer coach compared to those with a nurse care manager [8]. A Cochrane review of 17 RCTs of peer support for a variety of chronic conditions showed small improvements in some measures, but not in hard clinical outcomes [9]. The 2 studies of diabetes in the Cochrane review showed reductions of HbA1c but not a significant difference between the intervention and control groups [10,11]. The peer-led Chronic Disease Self-Management Program improved care, compared with controls, for several outcomes in patients with a variety of chronic conditions [12]. The same program for Latino patients with diabetes found a significant reduction in HbA1c for intervention patients compared with controls [13].

The project described in this paper will add to the existing body of knowledge regarding the efficacy of peer coaching to improve clinical outcomes in patients with diabetes. Our aim is to show that peer coaching that provides self-management support in a primary care setting improves clinical outcomes in low-income patients with poorly controlled diabetes. In addition, the qualitative component of this study will characterize the perspectives of peer coaches in order to better understand the reality of peer health coaching in primary care, which is not currently described in the literature.

## Materials/Design

### Objectives

This study investigates whether peer coaching over a 6 month period improves clinical outcomes (HbA1c, low-density lipoprotein cholesterol, blood pressure, and BMI), medication adherence, reported self-efficacy, and self-care activities in poorly-controlled patients with diabetes. Furthermore, we examine the same outcomes in the peer coaches - all of whom have diabetes.

### Study design

This study is an unblinded randomized controlled trial. Patients are randomized to one of two arms: (1) peer health coaching from patients with diabetes who are trained as peer coaches or (2) usual care.

### Ethics

Approval to conduct this study is granted by the Committee on Human Research (Institutional Review Board) at the University of California, San Francisco (approval number H40013-34104-01-01).

### Study population and site

All participants are low-income English or Spanish speaking patients with diabetes who receive primary care at one of the following San Francisco Department of Health primary care health centers: (1) Castro Mission Health Center, (2) Maxine Hall Health Center, (3) Potrero Hill Health Center, (4) Southeast Health Center, (5) General Medical Clinic at San Francisco General Hospital, and (6) Family Health Center at San Francisco General Hospital. The study team collaborates with the staff at each clinic to implement and monitor the study.

### Eligibility criteria

#### Peer coaches

Potential peer coaches are patients with type 2 diabetes and HbA1c ≤ 8.5% who are able to manage their diabetes and demonstrate personality traits suitable for working with patients. Utilizing these criteria, the study team asked providers or other clinic staff for recommendations. Peer coach candidates are able to: read and write in English or Spanish, attend a 36-hour training, work with patients and track those encounters for at least 6 months, attend a monthly coach meeting, and demonstrate basic diabetes self management knowledge and supportive, non-judgmental communication skills.

#### Patients

Eligible patients have type 2 diabetes and HbA1c ≥ 8% in the past 6 months. Additionally, patients plan to reside in the San Francisco area during the intervention period, have access to a telephone, and speak English or Spanish. Patients with a life expectancy of less than a year, serious comorbidities or reduced cognitive capacity as determined by the patient's provider or those currently enrolled in a diabetes management program or study are not eligible for this study.

### Identification and recruitment of participants

#### Peer coaches

The San Francisco Department of Public Health's electronic patient registry, i2iTracks, is searched to identify potential peer coaches who: (1) are assigned to a San Francisco Department of Health Primary Health Center (2) have a diagnosis of type 2 diabetes, (3) a HbA1c ≤ 8.5% measured within the last 6 months, and (4) speak English or Spanish.

To assist with selection of potential peer coaches, primary care clinicians (or other clinic staff familiar with the patients) review the registry search results. Other potential coaches are identified by clinicians' and staff members' recommendations, from clinic diabetes classes and from posted flyers. The study team contacts potential coaches via letters and phone calls to explain the study and extend an invitation to a recruitment event in the clinic.

#### Patients

The electronic registry is searched to identify adult patients who: (1) are assigned to one of the six San Francisco Department of Health Primary Health Centers listed above, (2) have a diagnosis of type 2 diabetes, (3) have a HbA1c > 8.0% measured within the last 6 months, and (4) speak English or Spanish. Patients identified as good peer coach candidates are removed from the list of potential patients. Additional patients are selected from clinic diabetes classes and from posted flyers. Primary care clinicians review search results and exclude patients with: a life expectancy of less than a year, serious comorbidities, reduced cognitive capacity or those not appropriate to participate in the study for other reasons.

The study team contacts eligible patients via letters and phone calls to explain the study and arrange an enrollment appointment. Patients who do not respond to letters or calls are screened at their clinical appointments.

### Enrollment and randomization

#### Peer coaches

Eligible peer coaches are screened to ensure that they: (1) read and write in English or Spanish, (2) plan to reside in San Francisco and continue receiving care at one of the six participating clinics for 12 months, (3) have a telephone, and (4) are willing to attend a 36 hour training, work with patients and track encounters for at least 6 months, and attend a monthly meeting to discuss patients. Those who answer yes to all of the above are given IRB-approved informed consent for enrollment in the study. Each potential coach completes a baseline survey. In addition, the study team collects blood pressure, weight and height values following the same guidelines as described for patients.

#### Patients

Prior to patient enrollment, the study team creates a randomization system to ensure unbiased sorting of patients to each study arm: to receive peer coaching vs. to receive usual care.

A language-concordant study team member contacts eligible patients by phone one week proceeding mailings. Patients who do not respond to letters or phone calls are approached at their next scheduled clinical appointment. Eligible patients who opt to participate are screened to ensure that he/she: (1) is willing to work with a peer coach if randomized to the coaching arm, (2) plans to reside in San Francisco for the next 6 months, (3) plans to continue receiving care at his/her assigned clinic for 6 months, (4) has a telephone, and (5) is not currently enrolled in a diabetes management program or study. Patients who answer yes to all of the above are given IRB-approved informed consent for enrollment into the study.

All participants complete a baseline survey, available on request from the authors. The study team then measures the blood pressure, weight, and height for each participant. Blood pressures are measured in the left arm after sitting for at least five minutes. The study team member obtains two blood pressure readings (with an Omron Home Blood Pressure Monitor Model 711-AC) two minutes apart, then averages the two for a final measurement. If the two systolic readings differ more than 5 points, a third blood pressure reading is measured and averaged with the two previous readings. Weight is obtained with a calibrated, portable, digital scale (HealthOmeter). Height is obtained using a tape measure and architect's right angle triangle. Participants with missing HbA1C or LDL cholesterol values in the past 6 months receive a lab requisition form to perform laboratory testing for these values.

At the completion of the consent process, patients learn the randomly generated enrollment assignment to either the active or control group. Patients in the active group select a peer coach from a coach profile. Each enrolled patient is compensated $10.

The study team notifies the peer coach by phone and provides the coach with the patient's name and contact information.

### Peer Coach Training

Peer coaches attend 36 hours of training at the San Francisco General Hospital led by the study team. Training is conducted in English and Spanish using a curriculum developed by the study team. Curriculum modules include: working collaboratively with patients (coaches refer to patients as clients); basics of diabetes; knowledge of diabetes medications; recognizing medical "red flags" such as symptoms of hypoglycemia; and navigating the clinic and accessing community resources. Furthermore, peer coaches are trained to: interact with clients using active listening and non-judgmental communication, help clients with diabetes self-management skills, provide social and emotional support, assist with lifestyle change, and facilitate medication understanding and adherence. Peer coaches that demonstrate competency during the training and who pass both written and oral examinations are included in the study.

### Intervention

Patients in the active arm are paired with a peer coach for six months, receive phone calls from coaches at least twice a month, meet face to face at least twice, and if possible, are accompanied to at least one clinic visit. Additional calls and meetings are at the discretion of client and coach. Coaches document each encounter attempt and record: date, the nature of encounter (phone or in person), approximate duration of contact, and topics discussed.

The first encounter is a phone call during which coaches introduce themselves and begin to establish a relationship with the client before discussing diabetes. In subsequent interactions the coach and client discuss: current and target clinical values for HbA1c, LDL cholesterol and blood pressure; self management skills such as using a glucometer and appropriate strategies for hypoglycemia; taking medications as prescribed; and lifestyle changes around healthy eating, physical activity, and stress. Coaches and clients may also share stories about family, careers, and hobbies.

### Sample size calculation

Sample size and power calculations were performed for the main outcome of interest - difference in mean HbA1c levels - using effect sizes and standard deviations derived from multiple published trials of patient-education interventions. The recent meta-analysis by Deakin et al. of 11 such studies found a mean difference in HbA1c of 0.8% [14]. Analysis of Family Health Center patients with HbA1c values 8.0% or higher showed a standard deviation of 1.9. Using a conservative assumption of an effect size of 0.6 for percent HbA1c, we would need 180 subjects in each arm to achieve a power of .85 using the standard threshold for a significant difference of .05 (2-sided). To account for a 10% loss to follow-up, we will enroll 200 subjects in each arm.

### Data analysis

Initial analyses will compare the frequency of baseline levels of outcome and other key variables (e.g., demographic and disease characteristics) for the two groups using a simple chi-square test (for categorical variables), t-test (for continuous variables with an approximately normal frequency distribution) and the Mann-Whitney-U test for continuous, non-normally distributed variables. Evaluation of intervention effectiveness will be by intention to treat using the above statistical tests. Evidence of clustering by clinic site and primary care provider will be examined and adjusted for analyses as needed. If significant differences in baseline characteristics are found, analyses will be repeated adjusting for these differences using ANOVA and logistic regression for multivariate analyses. Sensitivity analyses will be performed to estimate the effects of missing data using different assumptions (e.g., imputed values). Additional analyses will be conducted to look for evidence of effect modification by pre-specified subgroups: baseline HbA1c (< median, > median) and by English as primary language (Y/N).

### Outcome measures

Outcomes variables are measured at baseline and at 6 months. Peer coaches are assessed at 12 months. The primary outcome is change in HbA1c. Secondary outcomes include change in: systolic blood pressure, BMI, LDL, diabetes self-care activities, medication adherence, diabetes-related quality of life, diabetes self-efficacy, and depression.

### Qualitative evaluation

The peer coaches will voluntarily participate in a focus group, semi-structured interviews, or both, to assess how they experienced the training and coaching process. The focus group is designed to reveal the coaches' general attitudes and facilitate the development of the interview guide. Semi-structured interviews, between 40-75 minutes in length, are conducted in either English or Spanish. Coaches are prompted to discuss their efficacy as coaches, the training experience, the impact of coaching on their management of their own diabetes, and their role in the health care team.

The focus group and interviews are audio recorded and transcribed. Using methods based on grounded theory, as described by Miles and Huberman, transcripts are encoded and organized to identify, develop and analyze themes [15].

## Discussion

Self-management training and support is an effective component of care for people with diabetes. Due to a shortage of time and resources, this critical support is not consistently delivered in most health care settings. The current study uses trained patients to provide this support. Facing the same struggles as the patients they coach, trained peer coaches can assist the health care team to deliver the recommended National Standards for Diabetes Self-Management Education [16].

In our study, we pair trained patients with better-controlled diabetes and patients with poorly controlled diabetes to provide one-on-one self-management support. Coaches are trained to: provide information about diabetes and healthy lifestyle choices pertaining to physical activity and nutrition; help patients with medication understanding and adherence; and work collaboratively with patients to create actions plans around behavior and lifestyle changes. Coaches and patients speak bi-weekly, meet face-to-face at least twice during the course of the 6 month intervention, and coaches are encouraged to attend the patient's clinical visit.

We hypothesize that patients in the intervention arm with a peer health coach will show significant improvements in the clinical indicators pertinent to diabetes (HbA1c, LDL cholesterol and blood pressure), diabetes self-efficacy, and self-care activities compared to patients in the control arm. In addition, we anticipate that trained peer coaches will demonstrate similar significant improved outcomes.

## Competing interests

The authors declare that they have no competing interests.

## Authors' contributions

AG, TB, EC and DT conceived the study. DT planned the statistical analysis and conducted sample size calculation. AG drafted the manuscript. All authors revised and approved the final manuscript.

## Pre-publication history

The pre-publication history for this paper can be accessed here:

http://www.biomedcentral.com/1471-2458/11/208/prepub
